# Family caregiving and chronic illness management in schizophrenia: positive and negative aspects of caregiving

**DOI:** 10.1186/s40359-022-00794-9

**Published:** 2022-03-31

**Authors:** Man-Man Peng, Zhiying Ma, Mao-Sheng Ran

**Affiliations:** 1 Institute of Advanced Studies in Humanities and Social Sciences, Beijing Normal University at Zhuhai, Zhuhai, 519087 China; 2 Crown Family School of Social Work, Policy, and Practice, The University of Chicago, Chicago, IL USA; 3 Department of Social Work and Social Administration, University of Hong Kong, Hong Kong SAR, China; 4 Mental Health Center, West China Hospital, Sichuan University, Chengdu, 610041 Sichuan China

**Keywords:** Schizophrenia, Long term care, Rural Mental Health, Family caregiver

## Abstract

**Background:**

We aimed to explore the long-term caregiving experiences of family caregivers of people with schizophrenia (PwS) in terms of both positive and negative aspects.

**Method:**

Utilising a purposive sampling method, we conducted in-depth interviews with 20 family caregivers of persons who had suffered from schizophrenia for more than 20 years. We empirically investigated their retrospective experiences of caregiver-patient interactions during a long period of family caregiving. We audio-recorded and transcribed the interviews into text. We thematically coded and analysed the transcribed text using a four-phase method of theme development.

**Findings:**

Schizophrenia might not only generate a caregiving burden, affect caregivers’ psychological status, and accordingly influence their coping strategies, but also have short- or long-term patient-related consequences.

**Discussion:**

Family caregivers should develop their stress management skills to cope with relevant life changes and increase their knowledge of the potential psychological consequences for care recipients resulting from negative caregiving strategies during home-based practice. Care recipients with schizophrenia in a relatively stable status should be empowered to take care of themselves. More effective family-based interventions for psychiatric nursing or individualised training for symptom management should be tailored to serve families’ diverse needs.

## Introduction

Schizophrenia is one of the most debilitating, costly adult mental disorders, afflicting approximately 7 out of 1000 people aged 15 to 35 [1, 2]. As a typical severe and persistent mental disorder, schizophrenia is a great challenge not only for the patient and his/her family, but also for society as a whole [3, 4]. Although the prevalence of severe mental disorders is high worldwide, health system resources have not yet sufficiently responded to the burden of mental disorders [5].

A meta-analysis showed that the prevalence of schizophrenia is 4.44 per 1000 people, and that the overall lifetime prevalence is 4.73 per 1000 people in rural China [3]. The shortage of medical resources and public health services is more common in rural areas than in urban ones [6, 7]. Given that community-based care is poorly developed in rural China, the majority of rural families choose to take care of people with schizophrenia (PwS) at home [8–10]. A prior empirical study found that the absence of a family caregiver predicts a poorer long-term outcome of PwS in rural communities [11]. Family caregivers play a crucial role in mental health care in the social context of rural China. It is a strategic intervention for authorities to support persons with serious mental disorders by empowering families and caregivers, especially in underdeveloped regions [10, 12]. Nevertheless, little is known about the specific needs of families with relatives suffering from chronic schizophrenia.

Cultural values and traditional beliefs exert a deep-rooted influence on people’s beliefs about providing care to an ill family member in a Chinese family [7]. Spouses, parents, and offspring play an irreplaceable role in providing life support to PwS. However, the reality of limited resources and support increases the difficulties of family caregiving in underdeveloped areas. Families of PwS suffer from a heavy burden, and family caregivers face the challenge of balancing caretaking responsibilities with their own lives [13]. They may experience a wide variety of caregiving burdens in terms of financial [6, 14, 15], physical [16, 17], mental [15, 18, 19], daily life [7, 13], and social aspects [20, 21]. For instance, previous literature has documented different types of psychological distress among family caregivers of PwS such as perceptions of guilt, a sense of disgrace/shame (also described as ‘losing face’), embarrassment, grief, worry, and resentment [17, 19]. Nevertheless, the voices of family caregivers who have been taking care of PwS for a long time in rural Chinese communities often go unheard by researchers and policy-makers. Most previous research has focused on the effects of schizophrenia on family caregivers and produced relevant empirical evidence such as on caregiving burden, psychological health, and quality of life [22–26]. However, reciprocal effects between PwS and their primary caregivers over the long-term course of chronic illness are less frequently discussed or investigated in the domain of schizophrenia caregiving.

This study was informed primarily by the theoretical paradigms of the theory of stress and coping (TSC) [27] and the theory of symptom management (TSM) [28]. The framework of TSC is applicable to explain individual differences during the process of family caregiving [27]. The Sociocultural Stress and Coping Model [29] involves cultural values in the domain of individualism and familism to interpret the differences and variations among persons with different cultural backgrounds. However, Knight and Sayegh (2010) argued that although the previous models have confounded ethnicity as a concept of culture with socioeconomic status, these theories may have neglected the notion that cultural values could potentially have a positive influence during the process of coping with caregiving stress [30]. Furthermore, the roles of positive appraisal, positive coping strategies, and contextual factors (e.g. caregiver characteristics) throughout the caregiving process should be considered during the family caregiving process [31]. However, current theories are insufficient to provide insight to comprehend caregivers’ long-term interactions with PwS in these aspects. In addition, the theory of symptom management provides a multidimensional model (i.e. the domains of personal, environmental, and health/illness) for understanding the management process of chronic ailments [28, 32, 33]. The emergence of psychotic symptoms in severe mental disorders is potentially affected by factors in environmental domains [28]. The majority of long-term illness management is carried out more frequently outside the formal health care setting, such as in conditions of home-based caregiving [34]). Nevertheless, little is known about the outcomes of the illness management process based on the context of the family itself, which is a crucial informal health care environment for PwS. The models derived from TSM are also unable to capture the dynamic, ongoing nature of symptoms throughout the trajectory of the disease [33]. Moreover, TSM has been widely used to explain the interactions between formal caregivers (e.g. nurses) and patients, yet little is known about how TSM can be applied to explore relationships between informal caregivers and PwS in home-based settings. In the field of mental health care, most existing theories have described a unidirectional process of family caregiving for care recipients with chronic illnesses. They are insufficient to provide a framework for understanding the possible reciprocal effects between family caregivers and PwS with respect to coping with caregiving burdens and managing the illness of PwS. Thus, previous theories need to be improved to provide a better understanding of caregiver–patient interactions during the course of chronic schizophrenia. Guided by these theoretical paradigms, we aimed to explore caregivers’ retrospective experiences of long-term interactions with PwS in terms of coping stress and the illness-management process.

## Methods

### Study design

We conducted a qualitative study to explore caregivers’ retrospective experiences of providing care and the dynamics of caregiver-patient interactions over a long period of family caregiving. We held face-to-face individual interviews with the family caregivers of PwS using a semistructured interview script, including several open-ended questions. The interview questions were focused on three aspects: (1) long-term caregiving strategies and patients’ reactions; (2) family caregivers’ caregiving-related encounters and responses to the caregiving burden; and (3) changes in the caregiver-patient relationship. After a pilot study, we adjusted or supplemented certain questions based on the patient-caregiver relationship and the actual situation when conducting the interviews. Qualitative interviews are conversations in which the interviewer may have a general plan of inquiry for direction and pursue particular topics based on the respondents’ answers [35]. This method is particularly advantageous for a complex response set given that family caregiving in schizophrenia is an intricate process in which family caregivers interact with PwS within diverse environments [36].

### Sample and participant recruitment

We recruited the participants (N = 20) from Chengdu, Sichuan, China. We sampled them from a secondary database of the Chengdu Mental Health Project (CMHP) in which PwS and their family caregivers have been investigated since the early 1990s. The CMHP is a longitudinal study on mental illness and mental health services in Xinjin District, Chengdu, where is a representative middle-income rural county in Southwest China [8, 9]. The participants were the current family caregivers of PwS who had been involved in two waves of CMHP surveys (1994 and 2015). The identifiers of potential participants were the household numbers and the names of the care recipients with schizophrenia. We utilised a purposive sampling method to identify information-rich cases and to deepen the understanding of the study’s theoretical framework.

We invited participants who met all of the following criteria to attend a face-to-face interview: (1) a family member of someone with a schizophrenia diagnosis in both 1994 and 2015 (selected from the CMHP database); (2) aged between 18 and 80; (3) provided a minimum of two hours of care daily to a care recipient with schizophrenia for no less than six months in the past year; and (4) able to speak and understand Mandarin or the Sichuan dialect. The criterion regarding caregiving hours was identical to that of a previous study. We established the age restriction because local caregivers older than 80 may find it too difficult to understand or answer interview questions. The exclusion criteria were as follows: (1) the caregiver had also been diagnosed with a mental disorder; (2) the caregiver was unable to communicate well; and (3) the care recipient had died. Ultimately, we recruited 20 family caregivers of PwS.

### Data collection and procedure

We collected the data in November 2018. First, following the aforementioned selection criteria, we created a potential participant list in which we preliminarily identified and selected approximately 50 suitable participants from the CMHP database. Then, we asked the administrative staff of a local hospital to help invite potential participants and to provide them with a brief introduction to the research content. Having contacted potential participants, we made a final list of interviewees. Before the interviews, we informed the potential participants of the study’s purpose, procedure, and possible risks and consequences of taking part. On the basis of voluntary and protection principles, the participants could decide for themselves whether to take part, and they could end the interview early if they felt uncomfortable. They consented to their participation and the audio-recording of the interview by signing applicable forms. Subsequently, we held an in-depth interview using a semistructured interview script for 45 min to 1 h with each participant in a meeting room at a local hospital or community health centre.

We collected sociodemographic information regarding each PwS and their family caregivers, including gender, age, employment status, education level, and marital status. We also obtained caregiving-related and household information, including caregiver-patient kinship, years of care provision, the living status of the person with schizophrenia, household living conditions, family size, and family income per month. The entire process was completely anonymous and confidential. We first transcribed the audio recordings of the interviews into electronic text to create a database. We then removed all identifiable characteristics (e.g. name, contact number, home address) from the transcripts. Our protocol was performed in accordance with the relevant guidelines and regulations by the Human Research Ethics Committee for Non-clinical Faculties, the University of Hong Kong. Informed consent was obtained from all subjects.

### Data analysis

The qualitative data were thematically coded using NVivo 12.0. Table 1 presents a codebook for analysing the qualitative data. We examined the transcripts using the four-phase method of theme development [32, 37] involving ‘initialisation,’ ‘construction,’ ‘rectification,’ and ‘finalisation.’ In the first phase, we carefully read the interview transcripts. We highlighted meaningful units and recurrent pieces of content, such as repeated events or thoughts, inconsistent expressions, and distinctive ideas. We then coded the raw data in line with the research questions. We also made reflective notes to record the analytical process for the convenience of follow-up data interpretation. In the second phase, we sorted and compared similar and different pieces of content from the transcripts. We labelled keywords and sentences on the basis of the themes’ meaning. In the third phase, we improved the accuracy of the previous coding by critically rechecking the themes and coding procedure, and carefully conducting an in-depth literature review. We further described and wrote down, in detail, connections between themes and subthemes to strengthen data saturation. In the final phase of data analysis, we developed a storyline to order the findings and to review the entire process, which was helpful in identifying any gaps in the data analysis.

**Table 1:** A codebook for the qualitative data

Themes	Subthemes
1. Long-term caregiving experiences	1.1 Caregiver transitions
	1.2 Medication supervision and assistance
	1.3 Daily care
2. Caregiving burden	2.1 Financial influences
	2.2 Daily housework and farming
	2.3 Psychological distress
	2.4 Stigma attached to schizophrenia
	2.5 Occupation choice and quality of life
	2.6 Social relationships and interactions
3. Positive thoughts	3.1 Satisfaction with one’s current life
	3.2 Feelings about the caregiver’s role
4. Coping strategies and patient reactions	4.1 Negative coping
	4.2 Positive coping and expectations for the future
5. Family roles and relationship restructuring	5.1 PwS as spouses: Spousal life
	5.2 PwS as parents: Parenting for the next generation
	5.3 PwS as adult children: Issues on taking care of the older generation

## Participants’ profiles

Table 2 summarises the sociodemographic traits of the participants and their care recipients. A total of 20 family caregivers participated in this qualitative study, of whom 12 (60%) were male and eight (40%) were female. The family caregivers ranged in age from 42 to 78, with an average age of 58.15 (SD = 11.65). The highest education level among the family caregivers was secondary school. The majority of the caregivers (90%) were married. The caregiver sample comprised two (10%) parental caregivers, 13 (65%) spousal caregivers, two (10%) adult–child caregivers, one (5%) sibling, and two (10%) people who were other relatives. Their years of care provision ranged from 20 to 38 years, with an average of 27.05 years (SD = 4.58).

**Table 2:** Demographic characteristics of the participants and caregiving-related information (N = 20)

Characteristics	n (%)/M (SD)	Characteristics	n (%)/M (SD)
**Caregiver’s gender**		**Patient’s gender**	
Male	12 (60.0)	Male	7 (35.0)
Female	8 (40.0)	Female	13 (65.0)
**Caregiver’s age (range: 42–78)**	58.15 (11.65)	**Patient’s age (range: 42–75)**	58.85 (11.30)
**Caregiver’s education level**		**Patient’s education level**	
Uneducated	3 (15.0)	Uneducated	4 (20.0)
Primary school	10 (50.0)	Primary school	9 (45.0)
Secondary school	7 (35.0)	Secondary school	6 (30.0)
High school or vocational school	0 (0)	High school or vocational school	1 (5.0)
**Caregiver’s marital status**		**Patient’s marital status**	
Single	0 (0)	Single	2 (10.0)
Married	18 (90.0)	Married	14 (70.0)
Divorced	1 (5.0)	Divorced	3 (15.0)
Widowed	1 (5.0)	Widowed	1 (5.0)
**Caregiver’s employment status**		**Patient’s employment status**	
Unemployed	3 (15.0)	Unemployed	13 (65.0)
Farmer	7 (35.0)	Farmer	5 (25.0)
Being a farmer and a part-time worker	5 (25.0)	Being a farmer and a part-time worker	0 (0)
Worker	4 (20.0)	Worker	2 (10.0)
Retired	1 (5.0)	Retired	0 (0)
**Caregiver’s relationship to the patient**		**Patient’s living status**	
Parent	2 (10.0)	Living alone	2 (10.0)
Spouse	13 (65.0)	Living with the family/caregiver	18 (90.0)
Child	2 (10.0)	**Household living conditions**	
Sibling	1 (5.0)	Village house	13 (65.0)
Other relatives (e.g. son-in-law, sister-in-law)	2 (10.0)	Resettlement housing	7 (35.0)
**Years of care provision (range 20–38)**	27.05 (4.58)	**Family size (range: 2–6)**	3.45 (1.23)
		**Household income per year**^**a**^** (CNY, range: 6000–84,000)**	28,620 (22,859.01)

*M*  mean, *SD *standard deviation

^a^Approx. CNY to USD exchange rate in 2018 (6.93.57:1). According to the Chengdu Statistical Yearbook, annual income of rural households per capita is 28912.5 CNY in 2018. In this study, the household annual income of the investigated families is lower than the aforementioned value in the local area

Regarding the care recipients, seven (35%) were male and 13 (65%) were female. The age of the PwS ranged from 42 to 75, with an average of 58.85 years (SD = 11.30). The highest education level reached by PwS was high school. Over half of the care recipients (70%) were married. According to their caregivers’ reports, 13 (65%) of the PwS were unemployed, two (25%) were farmers, and two (10%) worked. Moreover, 13 (65%) were living in a village, and seven (35%) were living in resettlement housing. Family sizes ranged from two to six people, with a mean of 3.45 individuals (SD = 1.23). Instead of sharing a fixed amount, some of the caregivers reported a range of their family income per month. In general, the minimum self-reported monthly income of the families investigated ranged from 500 to 6000 CNY, with 2250 (SD = 1637.87) CNY on average, while the maximum ranged from 600 to 7000, with 2760 (SD = 2027.55) CNY on average.

## Findings

Five basic themes emerged in the family caregivers’ narratives: (1) long-term caregiving experiences; (2) caregiving burden; (3) positive thoughts; (4) coping strategies and the patient’s reaction; and (5) family roles and relationship restructuring. Each theme included several subthemes.

### Long-term caregiving experience

#### Primary caregiver transition

In the overall sample, 18 out of 20 participants (90%) had been serving as the primary caregiver since the patient’s first episode. The other two (10%) experienced a transition in their role: one brother’s wife became his primary caregiver 20 years before the interview, and one son had changed from being the secondary to the primary caregiver (one year before the interview) after the previous primary caregiver (the patient’s husband) had passed away.

Among the 20 families, there was a sequence in terms of the primary caregiver arrangement. Spouses or parents were usually expected to assume the main care responsibilities; in contrast, there was little expectation of siblings being the primary caregivers [38–43]. Moreover, children represented the last resort as primary caregivers in rural Chinese communities. In cases where the primary caregiver never changed, this was not due to a lack of desire. Rather, it was often difficult to find a new primary caregiver when considering how to prioritise caregiving responsibilities among family members.

‘My son? Everyone has a family. That is, when I went downtown [one time] for several days, it was still not easy to ask him to take care of his mother’. (No. 3, husband)

#### Medication supervision and assistance

In the interviews, we asked the family caregivers if the care recipients had been taking medication in recent years and whether they took medication on their own initiative or under their caregiver’s supervision. The majority of the caregivers (n = 18; 90%) reported that they often needed to supervise their care recipients and remind them to take medicine every day during treatment. Some PwS had a strong sense of self-stigma and refused to take medication. Some were willing to take medicine when they felt it worked. A small number of participants (n = 2; 10%) reported that their care recipients consciously followed the doctor’s advice and took their medicine on time. Thus, the care recipients adhered to their medical advice with the assistance of their family caregivers.

‘She takes the medicine twice a day. It is not difficult for her to take the medicine...She takes the medicine by herself, but I put it [in a bottle] for her every day. I put it in the bottle twice a day. I am in charge of managing her medication, including what kind of medicine to take every day, how much medicine to take every day, and how often to take the medicine. She takes it with a bottle in her hand, and I have to watch her to see if she takes it or not’. (No. 15, father)

#### Daily care

Most of the caregivers (n = 18, 90%) reported that their main role is to provide daily care to the PwS. In addition to medication supervision and assistance, they generally cook meals and complete housework for PwS, such as washing their clothes or cleaning the floor.

‘No, we are not divided. We usually ask our son to deliver the meal [to him] after we finish cooking. My son, who is in his thirties, often packs the leftovers up for his uncle [who has schizophrenia] when he eats out. I asked him, “Aren’t you being afraid of being laughed at by other people”? He said, “There is nothing to laugh about. Anyway, these leftovers would be thrown away [if I didn’t bring them to my uncle]”’. (No. 10, brother’s wife)

### Caregiving burden

#### Financial influences

The financial burden involved—especially in terms of expenses for medication—was most commonly mentioned by the family caregivers (n = 19; 95%) when discussing the difficulties of caring for PwS. Indeed, heavy financial burden generated some adverse psychological consequences for the families. It also affected family decisions in many aspects, such as opting against sending one’s children to school.

‘I suffered a lot in the early years [swearing]. At one point, we did not have any money and we did not eat meat for three to four months. At the time, I needed to buy him the medicine, but my family had no source of income. Nobody was earning money. My children needed to go to school, but we didn’t have money. I needed to pay several hundred yuan for their tuition fees. My older daughter was finally supported enough to go to university near our neighbours’. (No. 17, wife)

#### Daily housework and farming

Some caregivers (n = 14; 70%) described how the PwS had no self-care ability, so they personally needed to do all the housework alone. Consequently, some claimed to experience a heavy burden, balancing their daily caregiving affairs and their own work, whether on a farm or elsewhere.

‘In terms of housework and farm work, you know, if my mother had the ability to work, I could do much less work. For example, if my mother could do the cooking and cleaning, my father would be able to take care of the farmland. Then, I would be able to concentrate on my job, right? However, now I have to take care of her instead, right’? (No. 7, son)

Other caregivers (n = 6; 30%) claimed to feel less stressed when the PwS had self-care ability, as he/she could help with daily housework.

‘Now she can still cook. She had defrosted the meat at approximately 8 o’clock this morning when I came back. She steamed the rice and the dishes two hours before I returned. She knows how to do everything. She also put my clothes in the washing machine…If she stays at home, she cooks meals. Everything else is normal’. (No. 10, husband)

#### Psychological distress

Over half of the caregivers (n = 13; 65%) described feelings of psychological and emotional distress during the process of providing care. One wife mentioned the difficulties of providing long-term care to her ill husband. She reported how her husband suffers relapses nightly, causing her to cry. As a result, she said she often feels grief, worry, and resentment.

‘Sometimes I truly hate him…I am in a bad mood, and I truly hate him every time he scolds me. What I said is, “If it were not restricted by law, I truly would like to kill you”. That’s how I felt. However, I can’t do that. My heart wouldn’t allow me to do it. To tell you the truth, he’s still alive and kicking. You can’t kill a living man, although you may think about doing so. No way. The law doesn’t allow you [to do that], does it’? (No. 18, wife)

One husband felt stressed because his ill wife could not share the housework and was unable to communicate effectively with him. He usually chose to control his negative emotions to protect her feelings. However, suppressing his emotions made him feel more stressed and miserable.

‘She’s very irritable. She often scolds me, but I still talk to her with a smile. I only present a smiling face, but actually I feel like a knife is stabbing me in the heart’. (No. 14, husband)

#### Stigma attached to schizophrenia

Some family caregivers (n = 8; 40%) reported feelings of disdain from others, as well as in their own personal thoughts associated with the stigma of schizophrenia. Caregivers who perceived a stigma against severe mental disorders felt like they were in a state of emotional distress [18, 20].

‘To be honest, as a man, sometimes I pity myself, but I don’t dare tell others [that my wife has schizophrenia]. If you speak out, people will laugh at you and say, ‘What have you been doing’? When I think about this, I still feel a little guilty. However, the disease is a fact, and there is nothing else that can be changed’. (No. 12, husband)

#### Choice of occupation and quality of life

The family caregivers mentioned the impacts of caring for PwS on their occupation and quality of life. Some (n = 8; 40%) expressed how their occupational choices were limited due to the caregiving tasks required, thus reducing their household income and negatively affecting their quality of life.

‘Although you might not find a good job, at least you could go out to work. [If] you could go out to work all year round, your living conditions would be much better, right? It would be much better. Somebody else eats meat every day, but I can only drink water. That’s a simple truth, right’? (No. 7, son)

#### Social relationships and interactions

Some caregivers (n = 8; 40%) reported problems in their interpersonal ties, such as avoiding interactions with neighbours for fear of experiencing discrimination, or worrying about PwS having conflicts with others. To avoid contact with non-family members, some refused to move from their dispersed village setting to a more concentrated resettlement community because they were worried that a crowded environment would induce troublesome behaviour in the care recipients. Some relevant concerns include the following:

‘A few years ago, when people in our village did not move away, she liked burning other people’s haystacks. It got much better after others moved away...In the past, [she] always aroused suspicion in others. Before they moved, seven to eight years ago, she injured her head with a knife. If we were to also move to the community, there would be many people around, interacting a lot with each other. We were afraid that she would arouse suspicion in others again, so we didn’t move’. (No. 7, son)

The degree of interaction with the neighbourhood and the burden of social relationships differed among the families. Some caregivers (n = 6; 30%) indicated that they enjoyed good relationships with their neighbours because the patient did not exhibit any troublesome behaviours.

‘She visits other people’s houses, sometimes here and sometimes there. She sometimes chats with others. She’s not annoying, you know? Some people still play with her. She’s not raving’. (No. 6, son-in-law)

## Positive thoughts

### Satisfaction with current life

#### Satisfaction with one’s current life

Compared to the past, some caregivers claimed to be satisfied with their lives. With decreasing family demands, the caregivers perceived less stress. Indeed, as the extra financial burden (e.g. educational and living expenses) diminished as their children grew up, family caregivers felt less stressed because it was now easier to balance different family roles and to deal with multiple household tasks.

‘Now I think I have a lot more money on hand than I used to because I have brought up my daughter…She doesn’t give me any family allowance right now, but she just got married, and I don’t want her to give me anything…Compared to past years, it’s much better now. The key point is that I had to pay her tuition fees previously. It cost 60,000 to 70,000 CNY [≈8669–10,114 U.S. dollars] to [let her] go to university for several years’. (No. 9, husband)

#### Feelings about the caregiver’s role

Positive thoughts about family caregiving were also reported while discussing the long-term caregiver’s role. Influenced by the cultural value of familism [6, 8], some caregivers viewed the family as a unit and family members as integral parts. Accordingly, they claimed that family members are expected to take care of each other.

‘I don’t have any feeling [about his illness]. Family members should take care of each other for whatever they need. They are siblings only for this lifetime. They may not have this fortune in their next life. So [we] should take care of [him] when needed’. (No. 10, brother’s wife)

### Coping strategies and patients’ reactions

#### Positive coping and expectations for the future

Most of the caregivers utilised adaptive coping strategies such as consulting psychiatrists, seeking help from social resources, and proactively communicating. Some caregivers expressed their expectations for the future in a positive way. For instance, one father believed that his ill daughter would be able to take care of him and his wife when they are old.

A few years ago, she [daughter] told me that I would be in good health and live for some years longer. She would be less trouble. My daughter is clearheaded. She also told us that when we get older, she will wash our clothes for us and help us with this and that. So you can see, she was like this when she was good’. (No. 15, father)

Furthermore, a husband thought that buying social insurance for himself and his ill wife would benefit them later in life.

‘I have already bought endowment insurance for myself. If our land is expropriated by the country [and we get the compensation], I will be able to buy social security insurance. I will take 1000 to 2000 CNY [≈145 to 289 U.S. dollars)] per month, which is enough to maintain my everyday lifestyle. As for her, I can afford to buy some [insurance] for her now. She will take the money when she is at a certain age. What I bought for her is disability insurance’. (No. 19, husband)

#### Negative coping and patients’ reactions

Whenever a PwS relapsed, some of the caregivers employed negative coping strategies instead of seeking help from professionals. For instance, one caregiver self-reported that he scolded and beat his ill wife when she relapsed or failed to do the housework.

‘I haven’t taken her to [the hospital]. I just shouted at her but she didn’t run away. Sometimes I was displeased: I beat her so that she would not dare [run away] again…I had a bad temper when I was younger. Sometimes when I went back [home] and if I saw that she hadn’t cooked a meal, I yelled at her. Sometimes when my mouth went dry and I had no idea [how to deal with anger, I would hit her a few times’. (No. 12, husband)

In another case, a father adopted totally different strategies to deal with his ill (and now deceased) son and ill daughter because he thought that the male patient was more violent and intractable.

‘Sometimes [we] used a stick to beat [him], sometimes. Once, their mother used a stick as thick as a bamboo pole to hit his skull…We don’t beat her [the daughter with schizophrenia]. Why don’t we beat her? When she relapsed, she was not as fierce as her older brother. Her older brother even hurt others after he relapsed. Unlike him, she just broke things. Her older brother hurt others but she did not, so we did not beat her’. (No. 15, father)

### Family role and relationship restructuring

#### The PwS as a spouse: spousal life

Among the 13 spousal caregivers, five (four wives and one husband) reported that although they still took care of and lived with their spouses with schizophrenia, they had slept separately for many years. In the case of the husband, although he still lived with his ill wife, they were separated from each other in daily life, and the husband only worried about his wife when she went missing. Meanwhile, all four wives expressed that they had no alternative but to accept their situation after marriage. Suffering from the contradictions between ethics and personal stress, they viewed having a husband suffering from a severe mental disorder as a misfortune. They stated:

‘I am so unfortunate to have met this person. People do not say so anymore, but when I first came [to his home], those neighbours knew about his illness and said, “You are too naïve. Why would you marry this kind of person”? I said, “What else can I do? This is my destiny. He is ill. If you leave him, who can take care of him”? So I took care of him by myself. This is my destiny. I didn’t do well in my previous life, but I will do better in my next life’. (No. 17, wife)

Another family tried to work out an alternative relationship. An ex-husband reported that with the agreement of his mother-in-law, he and the patient could legally obtain a divorce certificate, but he promised to keep taking care of her. He tried to date other women and expected them to take care of his ex-wife.

‘This was agreed upon by my mother-in-law. When my son was older than ten, I was thinking about marrying another person, but I would primarily take care of her. I did have one [girlfriend] before. However, she did not want to stay with me because I was still taking care of her’. (No. 20, ex-husband & cohabitant)

#### PwS as parents: parenting for the next generation

Some family caregivers suggested that the PwS failed to parent and educate their children. As a result, while taking care of the PwS, these caregivers also took over responsibilities for parenting the PwS’ children. For instance, one sister served as the primary caregiver, not only for her younger brother with schizophrenia, but also for his son (her nephew).

‘I have been taking care of him [my nephew] for more than 20 years…I have been taking care of his [my brother’s] son all the time [since he was born]. He ate the milk powder that I bought. I brought him up…[My brother with schizophrenia] never took care of his son. [He] wasn’t concerned about [his son] at all’. (No. 2, older sister)

#### PwS as adult children: Taking care of the older generation

Influenced by cultural norms in Chinese society, relative to daughters, sons are generally more expected to take over the main responsibilities for caring for ageing parents. One sibling mentioned that she needed to take care of the older generation on her own, while the PwS failed to help due to their illness. In one case, a sister expressed a great degree of psychological strain in terms of taking care of her ill brother and her ageing mother after starting a family of her own.

‘You know, my father died early. My mother is getting old, and she’s exhausted [from all the years of caregiving]. She has to live [with me]. What else can she do? I also needed to take care of his [my brother’s] son. I took his son with us whenever we drove the truck to the market’. (No. 2, older sister)

## Discussion

To the best of our knowledge, this study is the first attempt to explore the long-term caregiving experiences of family caregivers who have been taking care of PwS for more than 20 years. This study deepens the understanding of interactions that take place between PwS and their family caregivers across the trajectory of the illness.

In our sample, the care recipients had been diagnosed with schizophrenia 24 years before the current study. When conducting this study, the family caregivers had been providing care to the PwS for an average of 27.05 years. More specifically, except for two cases (a son and a sister-in-law) where the respondent had changed from the role of secondary to primary caregiver prior to this study, 19 family caregivers reported having been the primary caregiver of a PwS for more than 20 years. This is congruent with previous literature documenting that family members play an irreplaceable role in providing life support to PwS, especially the patients’ spouses, parents, or descendants [44, 45]. Unlike individualism in Western societies, familism is a significant cultural value in rural Chinese communities that fundamentally affects the thoughts and actions of caregivers in caring for PwS [6, 20, 46]. Therefore, in rural Chinese communities, people view the family as a unit and family members as integral parts; thus, family members are usually expected to take care of each other. Consistent with previous research revealing poor medication adherence among PwS [21, 46, 47], most of the caregivers reported needing to supervise their care recipients and having to remind them to take medicine every day. Some of the care recipients perceived that mental disorders were stigmatised and consequently refused to take medication, especially when they believed the medicine had no significant effects. In rural Chinese communities, influenced by a stigma regarding severe mental disorders, some family caregivers may prefer to keep PwS at home and avoid exposing them in public. This is consistent with the findings of a prior study conducted in the Chinese social context in which family caregivers of PwS self-reported that they chose to lock their ill members at home and not allow them to attend outdoor activities for fear of ‘losing face’ [20]. The ways in which internalised stigma and social discrimination have long-term impacts on medication adherence and symptom management among PwS in rural Chinese communities require further research. When the PwS had no self-care ability, the caregivers perceived a great degree of psychological distress and found it difficult to balance their caregiving tasks with farm work or other jobs. Therefore, encouraging patients to become involved in the process of symptom management and empowering them to take care of themselves is essential [48, 49].

The most frequently perceived impact of care provision was the burden of caregiving. In addition to providing daily care, some caregivers suppressed their negative feelings for fear of affecting their care recipient’s emotional status, but this led to a greater degree of psychological distress. This is what sociologists call ‘emotional labour’ [50]: Caregivers learn to manage their experiences and expressions of emotions when interacting with the patient in order to perform their role as a caregiver or family member more effectively, but doing so takes a toll on them. Moreover, in line with previous research indicating the financial impacts posed by schizophrenia [6, 14, 15], the financial burden not only increased the degree of psychological strain among family caregivers, but also affected family decisions in many regards. Certainly, the family caregivers in this study reported facing difficulties in coping with their relationships with neighbours due to the stigma of mental disorder, or out of fear that the PwS would exhibit troublesome behaviour outside the family. This finding is in line with past studies documenting a strong sense of social isolation or a considerable degree of pressure on social life and social interactions among caregivers of PwS [7, 14, 15, 19]. Hence, identifying caregivers’ external and internal stressors is crucial to understanding their needs.

We found new evidence that the cultural value of family harmony and cohesion positively affects family members in terms of motivating them to look after PwS in rural Chinese communities [6, 8]. For instance, the caregivers mentioned that family members are supposed to take care of each other. This finding resonates with a recent systematic review showing that the positive impacts of schizophrenia on family caregivers include helping them recognise the values of family solidarity, compassion, and affection [51]. Further, due to the illness, family relationships are often restructured, potentially influencing spousal life, generating issues in parenting the next generation, or implicating caregiving for the older generation. Some people might even break away from the traditional family structure, such as in case where a husband gained permission from his mother-in-law to legally divorce his wife with schizophrenia as long as he continued acting as her primary caregiver.

The framework of Stress and coping theory [27] helps to explain individual differences during the process of family caregiving. According to this theory, when confronted with a stressful situation, an individual might use positive or negative coping mechanisms to adjust his/her psychological status or to reduce adverse consequences [52, 53]. In a positive light, most caregivers in this study utilised adaptive coping strategies, such as proactive communication with their care recipient, consulting professionals, seeking help from social resources, making a retirement plan, and purchasing medical insurance. In a negative light, some caregivers resorted to scolding and beating their care recipient to deal with stressful encounters. To prevent the exacerbation of schizophrenia in family caregiving, regular stress-management training and family interventions should be tailored to serve family caregivers who have been taking care of PwS for a long period of time in rural Chinese communities.

### Implications for research, practice and social policy

First, the current study sheds light on the theoretical paradigm of the TSC and TSM by adding empirical evidence that chronic mental illnesses might not only generate a caregiving burden, affect caregivers’ psychological status, and accordingly influence their coping strategies [54–57], but also may have patient-related consequences in the short and long term. These consequences might be directly or indirectly caused by the behaviours or attitudes of family caregivers when providing care. The TSC was also improved by clarifying the differentiations of disease-related stressors for various caregiving roles (i.e. parental, spousal, children, and sibling caregivers, and caregivers who are other relatives). In addition, the present study strengthens the contextualisation and utility of TSM by describing the dynamic relationship between a care provider and a care recipient with a chronic type of schizophrenia in an informal health care setting. This study adds to the existing knowledge for understanding (1) the impacts of schizophrenia on different types of caregiving burden sorted by kinship type (i.e. parents, spouses, children, siblings, and other relatives); and (2) the impacts of changes in primary caregivers and the quality of family caregiving on PwS in rural Chinese communities. For future studies, the mechanism behind the interaction between family caregiving and the symptom status of PwS across the trajectory of the illness deserves further research.

Second, for home-based practice in the rural Chinese context, caregivers should become acquainted with stress-management skills to cope with life changes and uncertainty after the onset of a patient’s illness, as well as knowledge of the potential psychological consequences of negative caregiving strategies. Meanwhile, a patient with a relatively stable status should be empowered to take care of himself/herself by receiving guidance on (1) how to recognise the symptoms of a relapse [32, 33]; (2) how to utilise a wide range of approaches to manage one’s symptoms, such as taking medication and seeking help from a doctor, nurse, or therapist [58]; and (3) how to improve one’s own ability to manage fluctuations in one’s illness and unpleasant symptoms [48, 49]. Thus, more effective, family-based interventions for psychiatric nursing or individualised training for symptom management should be tailored to serve the diverse needs of the local families of PwS in rural Chinese communities.

Third, at the social policy level, current policies and the mental health care system need to be improved by actions such as strengthening the coordination of local hospitals and community-based rehabilitation services [10]. Alleviating the caregiving burden might be a direct approach to lessen stress among family caregivers for PwS [10]. The burden of schizophrenia on families could also be alleviated by assisting families in other ways, such as by lowering their financial burden through waiving medical expenses. Increasing provision and improving the accessibility of public resources for PwS in community-based settings warrants further investigation [59].

### Limitations and future studies

Several limitations of the current study should be mentioned. Since we selected the sample from an existing database and the participants were caregivers who had been taking care of PwS for over 20 years, sampling bias might exist. It might not be possible to generalise the findings to all family caregivers of PwS, such as secondary caregivers or caregivers in urban areas. Therefore, caution should be applied in interpreting the findings. The perspectives of families in which the primary caregivers have changed several times should be included in future investigations. Second, given that our main focus was on caregiver-patient interactions during a long period of family caregiving in both negative and positive aspects, more research is needed to explore other important concepts of the caregiving process, such as the concept of ‘normalisation’ and relevant gender differences [60]. Third, since we mainly investigated the retrospective experiences of primary caregivers, we did not capture the dynamics of the interactions between other family members and PwS. Future empirical studies should incorporate data on other family members and the PwS to explore the impact of changing family structures on the family, as well as the consequences for the patient of long-term caregiving strategies across the trajectory of the illness.

## Data Availability

The data are not publicly available due to restrictions of the material, which compromises the privacy of the participants.

## Abbreviations

WHO: World Health Organization
PwS: People with schizophrenia
CMHP: Chengdu Mental Health Project
